# The impact of completion time on the reliability and validity of online electronic questionnaires: a preliminary study of the FABQ and ODI scales

**DOI:** 10.3389/fpsyg.2026.1717264

**Published:** 2026-03-31

**Authors:** Shilin Bi, Chao Wang, Haowei Liu, Li Huang, Haodong Tian, Xing Zhang, Hansen Li

**Affiliations:** 1 Department of Physical Education, Xinjiang Hetian College, Xinjiang, China; 2 School of Physical Education, Sichuan Agricultural University, Ya’an, China; 3 Faculty of Psychology, Southwest University, Chongqing, China; 4 Department of Physical Education and Sport, Faculty of Sport Sciences, University of Granada, Granada, Spain

**Keywords:** health, investigation, psychology, questionnaire, research, survey

## Abstract

This study uses secondary analysis of a health survey dataset to explore the impact of completion time on the reliability and validity of online questionnaires. A total of 1,758 chronic low back pain patients recruited online were included in the study. Using two widely used scales for assessing low back pain, the Fear-Avoidance Beliefs Questionnaire (FABQ) and the Oswestry Disability Index (ODI), we analyzed internal consistency, structural validity, and convergent validity across different completion time groups. Multi-group analyses based on time quartiles revealed that completion time exerted some influence on the internal consistency and structural validity of the FABQ, though slower completion times did not consistently yield better results. However, the internal consistency and structural validity of the ODI remained relatively stable across the different completion time groups. Longer completion times were associated with greater response variability in the FABQ but not in the ODI (in unadjusted analyses). Among participants who completed the questionnaires more quickly (the first 50% for FABQ and the first 25% for ODI), convergent validity was less ideal, with some results being contrary to expectations (when examining the correlations with self-reported happiness, sleep quality, and pain levels). In conclusion, our study supports the use of completion time as an important indicator for assessing the quality of online survey data.

## 1 Introduction

Questionnaire-based surveys remain one of the most widely used approaches for collecting data in psychology, health, and behavioral research. However, the usefulness of survey findings depends fundamentally on data quality. Even when well-established instruments are used, results can be distorted if respondents complete questionnaires inattentively, rush through items, or provide low-effort answers. These concerns are particularly important because poor-quality responses may not only increase random error, but also weaken the reliability and validity of measurement results, thereby limiting the interpretability of subsequent analyses (Berinsky et al., 2014; Meade and Craig, 2012; Ward and Meade, 2023).

The growing digitalization of survey administration has made this issue even more relevant. Compared with traditional paper-based formats, electronic questionnaires administered via computers, tablets, or smartphones offer clear practical advantages, including lower administrative burden, fewer data-entry errors, faster recruitment, and wider geographic reach (Peer et al., 2022; Van Selm and Jankowski, 2006). At the same time, however, online surveys are often completed in relatively unsupervised environments, where respondents may be distracted, multitask, or disengage more easily than in controlled settings (Gottfried, 2024; Olbrich et al., 2025). Thus, the shift to online data collection has created a methodological tension: although digital survey administration improves efficiency, it may also increase the risk of careless or insufficient-effort responding.

Importantly, digital survey systems also provide new opportunities to monitor data quality. In addition to traditional screening approaches such as attention checks and response-pattern analyses, electronic platforms can automatically record completion time. Completion time is attractive as a potential indicator because it is unobtrusive, inexpensive, and readily available in most online survey systems. The underlying logic is straightforward: meaningful responding requires at least some minimum time to read, understand, and answer the items, and extremely rapid responding may therefore signal insufficient effort or superficial processing (Goldammer et al., 2020; Goldammer et al., 2024). Nevertheless, completion time should not be treated as a definitive indicator on its own, because response speed may also reflect reading ability, questionnaire familiarity, device type, cognitive efficiency, or other individual characteristics (Ward and Meade, 2023). For this reason, the key question is not simply whether respondents are fast or slow, but whether differences in completion time are actually associated with differences in psychometric quality.

Existing evidence on this issue remains limited. Some earlier studies have suggested that response speed may have little influence on internal consistency in certain online questionnaires (Harms et al., 2017; Montag and Reuter, 2008). However, reliability is only one aspect of measurement quality. Much less is known about whether completion time is related to broader forms of validity, such as structural validity and convergent validity, particularly in applied clinical self-report instruments. This gap is important because patient-reported questionnaires are often used to support substantive inferences about symptoms, disability, and psychological status. If unusually rapid completion is associated with altered factor structure or atypical relationships with external criteria, then completion time may provide useful additional information for evaluating the credibility of online questionnaire data.

This issue may be especially relevant in chronic low back pain research, where self-report instruments are commonly used to assess both psychological and functional aspects of the condition. In the present study, we focused on two widely used measures: the Fear-Avoidance Beliefs Questionnaire (FABQ), which assesses pain-related fear-avoidance beliefs, and the Oswestry Disability Index (ODI), which assesses disability related to low back pain. These two instruments are clinically important, widely used, and structurally different, making them useful examples for examining whether completion time has similar or scale-specific associations with psychometric performance.

Therefore, the present study examined whether questionnaire completion time was associated with differences in the psychometric performance of the FABQ and ODI in an online sample of adults with chronic low back pain. Specifically, we compared internal consistency, structural validity, and convergent validity across completion-time groups, and also explored the association between completion time and response variability. By doing so, we aimed to evaluate whether completion time may serve as an auxiliary indicator of data quality in online electronic questionnaires.

## 2 Research design

### 2.1 Participants

This study is based on a secondary analysis of a dataset generated from a previous health survey in Chongqing, China. The informed consent and ethical approval (Code: SWU-ETH-2023-09-12-002) were obtained before starting the survey.

The original survey recruited 1,758 participants from internet communities across China. The inclusion criteria were: (1) aged at least 18 and (2) having chronic low back pain for at least 3 months. The sample size is appropriate according to previous calculations (Liu et al., 2024).

As the core of the survey, the FABQ and ODI scales were independently timed (in millisecond) during completion, which provided the data necessary for this study. We configured each questionnaire as a separate page and used page-level paradata to capture completion time. Specifically, completion time was defined as the elapsed time from when a participant opened the questionnaire page to when they finished all items and the system automatically advanced to the next page.

### 2.2 Measurement tools

#### 2.2.1 Fear-avoidance beliefs questionnaire

The Fear-Avoidance Beliefs Questionnaire (FABQ) is a psychological measurement tool used to assess an individual’s fear and avoidance behaviors related to pain, commonly used in the evaluation of patients with low back pain (Pei et al., 2010; Waddell et al., 1993). The FABQ consists of two subscales: Physical Activity (FABQ-PA) and Work (FABQ-W), with a total of 16 items. Each item is rated on a 7-point Likert scale (1 = “strongly disagree,” 7 = “strongly agree”). Of the 16 items, 13 are scored and used for statistical analysis, while the remaining three items (8, 13, and 14) are used for auxiliary judgment. Higher total scores indicate higher levels of fear-avoidance.

#### 2.2.2 Oswestry disability index

The Oswestry Disability Index (ODI) is a widely used tool to assess the degree of functional impairment in individuals with lumbar spine disorders (Chow and Chan, 2005; Fairbank and Pynsent, 2000). The ODI consists of 10 items that assess aspects such as pain intensity, personal care, lifting, walking, sitting, standing, sleeping, sexual activity, social activities, and travel. The ODI is generally considered a unidimensional scale (Gabel et al., 2017). Each item offers six response options, and in this study, a 1–6 scoring system was applied, with higher scores indicating greater functional impairment.

#### 2.2.3 Sleep quality, subjective happiness, and pain severity

Participants’ sleep quality (Cappelleri et al., 2009; Snyder et al., 2018), subjective happiness (Abdel-Khalek, 2006), and pain severity at the affected site (Hawker et al., 2011) over the past week were measured using single-item 11-point response scales.

#### 2.2.4 Demographic characteristics

Participants’ demographic characteristics, including age, gender, education level, household income, and marital status, were assessed using a questionnaire (the classification methods are detailed in the Results section).

### 2.3 Statistical analysis

#### 2.3.1 Main analysis

Adopting the quartile-split approach used in previous studies to handle survey completion time (Hansen et al., 2023; Mayerl, 2013), we divided participants into four approximately equal groups based on their completion time for the FABQ and ODI, respectively (*n* = 439, 440, 440, and 439). We used this quartile-split strategy for two reasons. First, it ensured a similar sample size across groups, while also providing an adequate number of participants in each group for the subsequent confirmatory factor analysis (see the CFA section below for the specific requirements). Second, it guaranteed that the data from each group were independent and non-overlapping, thereby improving the interpretability of the results. Subsequently, separate analyses were conducted for each subgroup.

Given potential response differences across groups (i.e., differences in score values and score distributions), we also reported the median, quartiles, mean, standard deviation, mode, kurtosis, and skewness separately for each of the four groups.

We used Chi-square tests or Fisher’s Exact tests (when frequencies were zero) to compare the distribution of participants’ demographic variables across the time groups.

To evaluate internal consistency of the scale dimensions, we calculated Cronbach’s alpha. A Cronbach’s alpha value greater than 0.7 is generally considered to indicate acceptable internal consistency (Taber, 2018).

We applied Confirmatory Factor Analysis (CFA) to assess the structural validity of the measurement tools, using Structural Equation Modeling (SEM) with Maximum Likelihood (ML) estimation. According to the COnsensus-based Standards for the selection of health Measurement INstruments (COSMIN) guidelines, the sample size for structural validity analysis should be at least seven times the number of items in the scale (Mokkink et al., 2019; Prinsen et al., 2018). According to common rules of thumb in structural equation modeling, the sample size should be at least 10 times the number of parameters to be estimated (Bagozzi and Yi, 2012). Based on these rules, our sample size meets the analysis requirements.

We used the following fit indices and their accepted standards: χ^2^/df < 3; Standardized Root Mean Square Residual (SRMR) < 0.08; Normed Fit Index (NFI) > 0.90; Tucker-Lewis Index (TLI) > 0.90; Comparative Fit Index (CFI) > 0.90; Root Mean Square Error of Approximation (RMSEA) < 0.08 (Sathyanarayana and Mohanasundaram, 2024).

Convergent validity was assessed by examining the correlations between the measurement results and related standards (Chin and Yao, 2024). We performed Spearman’s rank correlation analysis to explore the relationship between the FABQ and ODI scores and sleep quality, happiness, and pain severity. Since both the FABQ and ODI measure negative conditions related to pain and disability, we hypothesized that they would be positively correlated with pain levels and negatively correlated with sleep quality and happiness.

As an additional analysis, we calculated the standard deviation for each participant’s responses to both scales, to measure the variability of responses. Finally, we examined the Spearman correlation between completion time and response variability.

#### 2.3.2 Sensitivity analysis

Considering the potential influence of participant characteristics on the response process, we additionally conducted general linear models. In these models, the total scores of the two scales and participants’ background characteristics were entered as independent variables, whereas happiness, pain, and sleep quality were treated as dependent variables. Gender and marital status were specified as factors, while age, income, and education level were included as covariates to estimate the overall average effects. To improve the robustness of the results, HC3 heteroscedasticity-consistent standard errors were applied in the regression analyses (Long and Ervin, 2000). Similarly, we also supplemented the analysis of the association between completion time and score standard deviation with general linear models, using completion time as the independent variable. Completion time was standardized before regression analysis to improve coefficient interpretability.

## 3 Results

### 3.1 Participant demographics

A total of 1,758 participants were included. Participant characteristics are summarized in Supplementary Table S1. Most participants were male and aged 26–40 years, had an undergraduate education, and reported a household income between 5,000 and 10,000 RMB. Some demographic distributions differed across completion-time quartiles; for example, participants aged 18–25 years were under-represented in the slowest ODI group (Group 4).

Completion time ranged from 4,685 to 667,950 ms for the ODI (mean = 38,358 ms [38.4 s], SD = 35,722 ms) and from 7,467 to 672,923 ms for the FABQ (mean = 39,750 ms [39.7 s], SD = 36,279 ms). Score distributions were broadly comparable across the four quartiles (Supplementary Tables S2, S3). For FABQ, mean (median) scores decreased modestly from Group 1 to Group 4 (56.51 [57] to 48.82 [51]). For ODI, Group 1 scored slightly higher than Groups 2–4, which were similar to each other.

### 3.2 Internal consistency

Internal consistency was acceptable in all groups (Table 1). For FABQ-PA, Cronbach’s alpha ranged from 0.781 (Group 1) to 0.919 (Group 2); for FABQ-W, from 0.875 (Group 1) to 0.939 (Group 2); and for ODI, from 0.877 (Group 1) to 0.934 (Group 2). Across both instruments, the fastest completion group (Group 1) consistently showed the lowest alpha, whereas Group 2 showed the highest.

**Table 1:** Cronbach’s alpha (internal consistency) for FABQ-PA, FABQ-W, and ODI across completion-time quartiles (1 = fastest, 4 = slowest; quartiles defined separately for each instrument).

Group	FABQ-PA	FABQ-W	ODI
1	0.781	0.875	0.877
2	0.919	0.939	0.934
3	0.859	0.894	0.900
4	0.838	0.895	0.888

### 3.3 Structural validity

For ODI, confirmatory factor analysis (CFA) fit indices were broadly similar across completion-time groups and were generally within acceptable thresholds (SRMR 0.030–0.043; CFI 0.954–0.972; RMSEA 0.051–0.080), with no clear trend by completion speed (Table 2). For FABQ, only Group 2 demonstrated broadly acceptable model fit (SRMR 0.025; CFI 0.974; RMSEA 0.076), whereas Groups 1, 3, and 4 showed poor fit (CFI 0.855–0.879; RMSEA 0.124–0.148). Notably, the two FABQ factors in Group 2 were highly correlated (>0.90), indicating limited discriminant validity in that subgroup. Notably, the χ²/df values were not ideal in all the groups.

**Table 2:** Confirmatory factor analysis (CFA) fit indices for the ODI one-factor model and FABQ two-factor model across completion-time quartiles (1 = fastest, 4 = slowest; quartiles defined separately for each instrument).

	Group	SRMR	χ^2^/df	NFI	IFI	TLI	CFI	RMSEA
ODI								
	1	0.032	2.152	0.950	0.972	0.964	0.972	0.051
	2	0.030	3.833	0.953	0.965	0.955	0.965	0.080
	3	0.043	3.430	0.938	0.956	0.9643	0.955	0.074
	4	0.037	3.220	0.934	0.954	0.940	0.954	0.071
FABQ								
	1	0.057	7.725	0.863	0.879	0.844	0.879	0.124
	2	0.025	3.550	0.965	0.974	0.967	0.974	0.076
	3	0.064	9.933	0.859	0.871	0.834	0.870	0.143
	4	0.066	10.637	0.843	0.856	0.815	0.855	0.148

### 3.4 Convergent validity

ODI scores were positively correlated with pain in all groups (Spearman’s rho = 0.129–0.399, all *p* < 0.01). Negative correlations with happiness and sleep quality were observed in Groups 2–4 (happiness rho = −0.165 to −0.262; sleep quality rho = −0.201 to −0.245; all *p* < 0.01), but not in Group 1 (Table 3). For FABQ, pain was positively correlated in all groups (rho = 0.287–0.442, all *p* < 0.01). However, correlations with happiness and sleep quality differed by completion speed: Group 1 showed counterintuitive positive correlations (happiness rho = 0.362; sleep quality rho = 0.424; both *p* < 0.01), Group 2 showed near-zero correlations, Group 3 showed a small negative correlation with happiness (rho = −0.141, *p* < 0.01) but not sleep quality, and Group 4 showed the expected negative correlations (happiness rho = −0.154, *p* < 0.01; sleep quality rho = −0.122, *p* < 0.05).

**Table 3:** Convergent validity: Spearman correlations between FABQ/ODI total scores and external criteria (happiness, sleep quality, pain) across completion-time quartiles (1 = fastest, 4 = slowest; quartiles defined separately for each instrument).

Group	Variable	Happiness	Sleep quality	Pain
1	FABQ	0.362**	0.424**	0.442**
2	FABQ	0.062	0.035	0.402**
3	FABQ	−0.141**	−0.051	0.287**
4	FABQ	−0.154**	−0.122*	0.370**
1	ODI	−0.061	0.017	0.129**
2	ODI	−0.262**	−0.245**	0.347**
3	ODI	−0.243**	−0.218**	0.399**
4	ODI	−0.165**	−0.201**	0.399**

*p < 0.05; **p < 0.01.

### 3.5 Relationship between completion time and response variability

Completion time was moderately associated with response variability for FABQ (rho = 0.418, *p* < 0.01), indicating greater within-person response dispersion among slower responders. No association was observed for ODI (rho = −0.002, *p* = 0.937).

### 3.6 Sensitivity analysis

In adjusted linear models (covariates: age, sex, marital status, education, and income; HC3 robust standard errors), higher FABQ scores were consistently associated with greater pain in all groups (*B* = 0.043–0.078, all *p* < 0.001). Associations with happiness and sleep quality remained completion-time dependent: FABQ was not associated with either outcome in Group 2 (*p* = 0.635 and 0.795), whereas significant negative associations were observed in Groups 3–4 (happiness *B* = −0.021 to −0.038; sleep quality *B* = −0.027 to −0.037; *p* ≤ 0.026) (Supplementary Table S4).

For ODI, higher scores were consistently associated with greater pain in all groups (*B* = 0.040–0.115, *p* ≤ 0.003). ODI was also negatively associated with happiness and sleep quality in Groups 2–4 (all *p* < 0.001), whereas these associations did not reach significance in Group 1 (happiness *p* = 0.089; sleep quality *p* = 0.635) (Supplementary Table S4).

After covariate adjustment, completion time remained positively associated with response variability for FABQ (*B* = 0.294, 95% CI 0.147–0.442; *p* < 0.001). For ODI, the adjusted association was statistically significant but small (*B* = 0.063, 95% CI 0.023–0.104; *p* = 0.002), differing from the null unadjusted correlation.

## 4 Discussion

### 4.1 General discussion

This study explored the impact of completion time on the validity and reliability of online electronic questionnaires, using the FABQ and ODI scales—two commonly used psychological measurement tools for patients with low back pain—as examples. Our findings suggest that completion time does influence the reliability and validity of the questionnaires to some extent, with particularly notable effects on convergent validity.

We found that completion time did not lower the internal consistency of the tools to an unacceptable level, which is consistent with previous studies (Harms et al., 2017; Montag and Reuter, 2008). However, it is important to note that very similar responses across items may artificially inflate internal consistency, which could give a misleading sense of reliability.

For the FABQ scale, the second fastest group (Group 2) exhibited the best internal consistency and structural validity (Tables 1, 2). However, we observed a significant positive correlation between completion time and response variability, suggesting that slower participants provided more diverse answers. This raises concerns that the seemingly better metrics for Group 2 might not be reliable. Confirmatory factor analysis revealed that the correlation between the two factors in Group 2 was too high (over 0.90), which is problematic as factor correlations are generally expected to be below 0.85 to indicate distinct dimensions (Ahmad et al., 2016). This suggests that participants in this group displayed overly similar response patterns across the two subscales. Furthermore, in the convergent validity analysis (Table 3), only Groups 3 and 4 showed results consistent with expectations and previous research findings (Chung et al., 2013; Kanaan et al., 2015). Meanwhile, the positive correlation between the FABQ and Happiness and Sleep Quality in Group 1 was counterintuitive, suggesting that the responses from this group were highly concentrated on one side of the scale (e.g., consistently high scores). One possibility is that very fast respondents did not carefully consider the meaning of each item, leading them to provide broad and highly consistent answers. The positive association we observed between FABQ completion time and response variance also suggests this possibility. Given that FABQ items are relatively simple and similar to one another—and therefore easier to answer—this may further increase the likelihood that some participants process the questionnaire superficially. In other words, because respondents may feel less concerned about giving obviously “wrong” answers, they may be more inclined to complete an easier-to-answer questionnaire in a more careless or rushed manner. Based on these findings, we conclude that fast completion of the FABQ likely leads to unreliable responses. Evidence from the convergent validity also suggests that participants who completed the questionnaire below the median completion time should be excluded.

In contrast, different results were observed for the ODI scale. First, the association between completion time and response variability was weak. A statistically significant association was observed only in the sensitivity analysis after controlling for participant characteristics, and the effect size was very small. The association between completion time and within-person response variance is likely scale-dependent. For the FABQ, items are brief Likert-type statements with overlapping content across two highly related domains (physical activity and work). Such a structure may be more susceptible to satisficing among very fast responders (e.g., using a global attitude or straightlining across similar items), yielding lower within-person response dispersion, whereas slower respondents may spend more time differentiating item content and thus show greater response variability. In contrast, the ODI uses more concrete, function-oriented items with comparatively longer response descriptions, which may impose greater reading and decision demands and reduce the opportunity for straightlining. This structural difference may explain why similar patterns among fast responders were observed for ODI but were smaller in magnitude than those for FABQ.

Second, internal consistency and structural validity indices were similar across groups and largely remained within acceptable ranges (Tables 1, 2). However, in the convergent validity analysis, the correlations between ODI scores and other variables (such as pain, happiness, and sleep quality) were consistently for Groups 2, 3, and 4, and were in directions that supported by previous research (Mannion et al., 2006; Zarrabian et al., 2014). In Group 1, however, the ODI scores were only positively correlated with Pain, suggesting that participants who completed the ODI very quickly (e.g., within the first 25%) may have provided problematic responses.

### 4.2 Limitations and future directions

Our quartile-based grouping is sample-dependent and may not generalize across populations or survey implementations. Quartiles provide a distribution-based approach that avoids arbitrary cut-offs, but the boundaries will shift with the sample’s demographic composition, survey context, and instrument characteristics. Consequently, the observed patterns across Groups 1–4 should be interpreted as relative differences within this dataset rather than universal thresholds that can be directly transferred to other settings.

As this study was a secondary analysis based on existing data, our choice of measurement tools was limited, and we only used the FABQ and ODI scales. The structural validity of the FABQ was found to be less than ideal. While this could be attributed to inherent issues with the tool itself [the original translation’s structural validity was similar to our observations (Pei et al., 2010)], it may also have limited the effectiveness of the convergent validity and internal consistency analyses (Pei et al., 2010). Additionally, because we focused on the impact of completion time on validity and reliability, we did not adjust the structural models as other psychological studies have done to improve fit indices (Acuña Mora et al., 2022; Andrea et al., 2024; Ibrahim et al., 2021).

In interpreting model fit across completion-time groups, we specified the CFA structure *a priori* based on the established literature rather than modifying the measurement model in response to the fit of any single subgroup. This decision was made to preserve cross-group comparability, as data-driven re-specification in one group (e.g., guided by modification indices) would imply a different measurement model and reduce the interpretability of between-group contrasts. However, this approach also has an important limitation. In one FABQ subgroup, the correlation between the two latent factors exceeded 0.90, indicating weakened discriminant validity. This pattern suggests that the hypothesized two-factor structure of the FABQ was not fully confirmed in our sample and that the distinction between the physical activity and work-related dimensions may have been less clear than theoretically expected in that subgroup. Although such a pattern was not observed in the other completion-time groups, and we therefore retained the conventional two-factor configuration used in prior validations of the Chinese FABQ, the FABQ-related findings should be interpreted with caution. While we treated suboptimal fit or cross-group discrepancies primarily as potential indicators of atypical response behavior or data quality limitations, we cannot fully rule out the possibility that the FABQ factor structure in our sample deviated from the original theoretical model.

Completion time may not solely reflect attentiveness or diligence; it can also index stable individual characteristics. For example, the manner in which participants complete questionnaires may partly reflect underlying personality traits (Furnham et al., 2013). In addition, demographic factors may shape response speed through differences in reading demands and cognitive resources: older adults and individuals with lower educational attainment may find the questionnaire more challenging and therefore take longer to complete, whereas younger respondents may complete the survey faster not because they are less attentive, but because higher cognitive efficiency allows quicker processing. Consistent with this possibility, we observed differences in social background characteristics across the completion-time groups. Although we adjusted for several key demographics (e.g., age, sex, marital status, education, and income) in sensitivity analyses, the impact of demographic factors may not be fully captured, and residual confounding remains possible because other relevant characteristics—such as health status, pain severity, digital literacy, reading comprehension, device type, and survey-taking context—were not directly measured. Moreover, demographic subgroups may differ not only in response speed but also in how items are interpreted (e.g., differential item functioning), which could contribute to heterogeneity in factor structure and validity patterns across completion-time groups. Future research should incorporate broader demographic and contextual measures and evaluate measurement invariance/DIF across key demographic groups to better disentangle demographic influences from completion-time effects.

Finally, our convergent validity evidence relied on a limited set of self-reported external criteria, and the study design was observational. Future research should incorporate richer paradata, objective or clinician-rated criteria where feasible, explicit data-quality indicators (e.g., attention checks), and formal invariance/DIF testing to strengthen the reliability of subgroup comparisons and improve external validity.

## 5 Conclusion

This study highlights the potential impact of completion time on the reliability and validity of online electronic questionnaires, providing important insights for improving data quality. The results show that for the FABQ scale, participants who completed the questionnaire too quickly (within the top 50% of fastest responders) exhibited anomalies in convergent validity, suggesting that their responses may need to be excluded during data cleaning. In contrast, the ODI scale was less affected by completion time, though discrepancies in convergent validity for fast responders remain noteworthy. We recommend that future questionnaire-based research consider incorporating both completion time and response variability as auxiliary quality control measures. This approach could help enhance the credibility of the data and the reliability of research conclusions, particularly in online surveys where monitoring participant engagement may be more challenging.

## Data Availability

The raw data supporting the conclusions of this article will be made available by the authors, without undue reservation.
