# Supplementary material for: The impact of completion time on the reliability and validity of online electronic questionnaires: a preliminary study of the FABQ and ODI scales
Source: Front Psychol. 2026 Mar 31;17:1717264. doi: 10.3389/fpsyg.2026.1717264 (PMC13076495; doi:10.3389/fpsyg.2026.1717264)
Supplement: Supplementary file 1 [file Table_1.docx]

**Supplemental Materials**

**Table 1. Participants’ characteristics**

| **Variable** | **Category** | **ODI group (N)** | | | | **FABQ group (N)** | | | | **Total (N)** |
| --- | --- | --- | --- | --- | --- | --- | --- | --- | --- | --- |
|  |  | **1** | **2** | **3** | **4** | **1** | **2** | **3** | **4** |  |
| Sex | Male | 359_a_ | 344_a_ | 341_a_ | 331_a_ | 359_a_ | 343_a, b_ | 354_a_ | 319_b_ | 1375 |
|  | Female | 80_a_ | 96_a_ | 99_a_ | 108_a_ | 80_a_ | 97_a, b_ | 86_a_ | 120_b_ | 383 |
| Age | 18-25 | 2_a_ | 4_a_ | 0_a_ | 0_a_ | 3_a_ | 2_a_ | 1_a_ | 0_a_ | 6 |
|  | 26-30 | 115_a_ | 108_a, b_ | 111_a_ | 76_b_ | 115_a_ | 115_a_ | 93_a_ | 87_a_ | 410 |
|  | 31-40 | 159_a_ | 128_a_ | 134_a_ | 150_a_ | 139_a_ | 152_a_ | 148_a_ | 132_a_ | 571 |
|  | 41-50 | 92_a_ | 146_b_ | 133_b_ | 166_b_ | 115_a, b_ | 102_b_ | 149_a, c_ | 171_c_ | 537 |
|  | 51-60 | 61_a_ | 47_a_ | 49_a_ | 40_a_ | 59_a_ | 59_a_ | 41_a_ | 38_a_ | 197 |
|  | 61-70 | 9_a_ | 6_a_ | 7_a_ | 7_a_ | 7_a_ | 8_a_ | 5_a_ | 9_a_ | 29 |
|  | >70 | 1_a_ | 1_a_ | 6_a_ | 0_a_ | 1_a_ | 2_a_ | 3_a_ | 2_a_ | 8 |
| Education | Middle school or lower | 10_a_ | 11_a_ | 8_a_ | 13_a_ | 10_a_ | 13_a_ | 10_a_ | 9_a_ | 42 |
|  | High school or equivalent | 64_a_ | 59_a_ | 85_a_ | 74_a_ | 77_a_ | 64_a_ | 65_a_ | 76_a_ | 282 |
|  | College degree | 144_a_ | 137_a, b_ | 104_b_ | 108_b_ | 145_a_ | 132_a, b_ | 104_b_ | 112_a, b_ | 493 |
|  | Bachelor Degree | 204_a_ | 218_a_ | 227_a_ | 235_a_ | 193_a_ | 209_a, b_ | 247_b_ | 235_b_ | 884 |
|  | Master or higher | 17_a_ | 15_a_ | 16_a_ | 9_a_ | 14_a, b_ | 22_b_ | 14_a, b_ | 7_a_ | 57 |
| Marriage | Unmarried | 160_a_ | 163_a_ | 148_a_ | 141_a_ | 154_a_ | 173_a_ | 146_a_ | 139_a_ | 612 |
|  | Married | 248_a_ | 269_a, b_ | 288_b_ | 295_b_ | 259_a_ | 255_a_ | 288_a, b_ | 298_b_ | 1100 |
|  | Divorced | 27_a_ | 8_b_ | 2_b_ | 3_b_ | 22_a_ | 12_a, b_ | 5_b, c_ | 1_c_ | 40 |
|  | Widowed | 4_a_ | 0_a_ | 2_a_ | 0_a_ | 4_a_ | 0_a_ | 1_a_ | 1_a_ | 6 |
| Income | <3000 RMB | 18_a_ | 13_a_ | 10_a_ | 10_a_ | 19_a_ | 19_a_ | 5_b_ | 8_a, b_ | 51 |
|  | 3000-5000 | 136_a_ | 115_a, b_ | 98_b_ | 85_b_ | 139_a_ | 108_a, b_ | 110_a_ | 77_b_ | 434 |
|  | 5001-10000 | 209_a_ | 183_a_ | 175_a_ | 190_a_ | 216_a_ | 208_a, b_ | 156_c_ | 177_b, c_ | 757 |
|  | >10000 RMB | 76_a_ | 129_b_ | 157_b_ | 154_b_ | 65_a_ | 105_b_ | 169_c_ | 177_c_ | 516 |
| **Variable** |  | **ODI group (mean & SD)** | | | | **FABQ group (mean & SD)** | | | | **Total (mean & SD)** |
|  |  | **1** | **2** | **3** | **4** | **1** | **2** | **3** | **4** |  |
| ODI time |  | 10287.79 (2434.74) | 22977.73 (4904.75) | 40724.66 (5203.81) | 79470.85 (48318.10) | - | - | - | - | 38357.85 (35722.45) |
| FABQ time |  | - | - | - | - | 12561.76 (1865.42) | 21860.60 (4222.00) | 41205.07 (6452.08) | 83409.45 (47344.80) | 39749.86 (36278.72) |
| ODI score |  | 31.53 (7.25) | 25.13 (8.75) | 23.10 (7.29) | 22.72 (6.90) | - | - | - | - | 25.62 (8.36) |
| FABQ score |  | - | - | - | - | 56.51 (10.06) | 53.85 (14.68) | 51.83 (12.34) | 48.82 (12.91) | 52.75 (12.91) |

**Note:** different lowercase letters indicate significant differences

**Table 2. Distribution of total FABQ score by group**

| **Statistic** | **Group 1 (n=439)** | **Group 2 (n=440)** | **Group 3 (n=440)** | **Group 4 (n=439)** |
| --- | --- | --- | --- | --- |
| Missing, n | 0 | 0 | 0 | 0 |
| Mean | 56.51 | 53.85 | 51.83 | 48.82 |
| Median | 57.00 | 56.00 | 53.00 | 51.00 |
| Mode | 60.00 | 77.00 | 57.00 | 51.00 |
| SD | 10.06 | 14.68 | 12.34 | 12.91 |
| Skewness | -0.82 | -0.92 | -0.54 | -0.57 |
| Kurtosis | 2.96 | 0.90 | 0.12 | 0.13 |
| 25th percentile (P25) | 50.00 | 48.00 | 44.00 | 40.00 |
| 50th percentile (P50) | 57.00 | 56.00 | 53.00 | 51.00 |
| 75th percentile (P75) | 63.00 | 64.00 | 61.00 | 58.00 |

**Table 3. Distribution of total ODI score by group**

| **Statistic** | **Group 1 (n=439)** | **Group 2 (n=440)** | **Group 3 (n=440)** | **Group 4 (n=439)** |
| --- | --- | --- | --- | --- |
| Missing, n | 0 | 0 | 0 | 0 |
| Mean | 31.53 | 25.13 | 23.10 | 22.72 |
| Median | 32.00 | 25.00 | 22.00 | 21.00 |
| Mode | 32.00 | 24.00 | 19.00 | 19.00* |
| SD | 7.25 | 8.75 | 7.29 | 6.90 |
| Skewness | -0.09 | 0.79 | 0.94 | 1.09 |
| Kurtosis | 3.21 | 1.27 | 1.38 | 1.64 |
| 25th percentile (P25) | 29.00 | 19.00 | 18.00 | 18.00 |
| 50th percentile (P50) | 32.00 | 25.00 | 22.00 | 21.00 |
| 75th percentile (P75) | 35.00 | 30.00 | 28.00 | 26.00 |

**Table 4. Associations between scale total scores (FABQ/ODI) and external outcomes across questionnaire completion-time groups**

**A) Predictor: FABQ total score**

| **Duration group** | **Outcome (DV)** | **Predictor** | **B** | **Robust SE** | **t** | **p** | **95% CI (Lower, Upper)** |
| --- | --- | --- | --- | --- | --- | --- | --- |
| 1 | Happiness | FABQ total | 0.053 | 0.012 | 4.521 | <0.001 | 0.030, 0.077 |
| 2 | Happiness | FABQ total | -0.003 | 0.007 | -0.474 | 0.635 | -0.018, 0.011 |
| 3 | Happiness | FABQ total | -0.021 | 0.009 | -2.231 | 0.026 | -0.039, -0.002 |
| 4 | Happiness | FABQ total | -0.038 | 0.007 | -5.180 | <0.001 | -0.052, -0.024 |
| 1 | Pain | FABQ total | 0.078 | 0.009 | 8.446 | <0.001 | 0.060, 0.097 |
| 2 | Pain | FABQ total | 0.047 | 0.008 | 5.574 | <0.001 | 0.030, 0.064 |
| 3 | Pain | FABQ total | 0.056 | 0.008 | 6.765 | <0.001 | 0.040, 0.073 |
| 4 | Pain | FABQ total | 0.043 | 0.007 | 5.783 | <0.001 | 0.029, 0.058 |
| 1 | Sleep quality | FABQ total | 0.070 | 0.013 | 5.351 | <0.001 | 0.044, 0.096 |
| 2 | Sleep quality | FABQ total | 0.002 | 0.008 | 0.260 | 0.795 | -0.014, 0.018 |
| 3 | Sleep quality | FABQ total | -0.027 | 0.009 | -2.932 | 0.004 | -0.045, -0.009 |
| 4 | Sleep quality | FABQ total | -0.037 | 0.008 | -4.413 | <0.001 | -0.054, -0.021 |

**B) Predictor: ODI total score**

| **Duration group** | **Outcome (DV)** | **Predictor** | **B** | **Robust SE** | **t** | **p** | **95% CI (Lower, Upper)** |
| --- | --- | --- | --- | --- | --- | --- | --- |
| 1 | Happiness | ODI total | -0.028 | 0.016 | -1.705 | 0.089 | -0.060, 0.004 |
| 2 | Happiness | ODI total | -0.085 | 0.015 | -5.724 | <0.001 | -0.115, -0.056 |
| 3 | Happiness | ODI total | -0.074 | 0.017 | -4.501 | <0.001 | -0.107, -0.042 |
| 4 | Happiness | ODI total | -0.060 | 0.018 | -3.345 | 0.001 | -0.095, -0.025 |
| 1 | Pain | ODI total | 0.040 | 0.014 | 2.994 | 0.003 | 0.014, 0.067 |
| 2 | Pain | ODI total | 0.084 | 0.011 | 7.876 | <0.001 | 0.063, 0.104 |
| 3 | Pain | ODI total | 0.115 | 0.011 | 10.371 | <0.001 | 0.093, 0.137 |
| 4 | Pain | ODI total | 0.106 | 0.011 | 9.947 | <0.001 | 0.085, 0.126 |
| 1 | Sleep quality | ODI total | -0.009 | 0.018 | -0.475 | 0.635 | -0.044, 0.027 |
| 2 | Sleep quality | ODI total | -0.082 | 0.016 | -5.128 | <0.001 | -0.113, -0.050 |
| 3 | Sleep quality | ODI total | -0.076 | 0.018 | -4.204 | <0.001 | -0.112, -0.041 |
| 4 | Sleep quality | ODI total | -0.083 | 0.019 | -4.329 | <0.001 | -0.121, -0.045 |
